# Learning Curve Analysis of Single-Site Robot-Assisted Hysterectomy

**DOI:** 10.3390/jcm11051378

**Published:** 2022-03-02

**Authors:** Yeon Jee Lee, Dong-Eun Lee, Jaekyung Bae, Hyeong In Ha, Myong Cheol Lim

**Affiliations:** 1 Center for Gynecologic Cancer, Research Institute and Hospital, National Cancer Center, Goyang 10408, Korea; gynyjlee@ncc.re.kr; 2 Biostatistics Collaboration Team, Research Institute, National Cancer Center, Goyang 10408, Korea; dong-eun@ncc.re.kr; 3 Department of Obstetrics and Gynecology, Seoul National University College of Medicine, Seoul 03080, Korea; 5d252@snuh.org; 4 Department of Obstetrics and Gynecology, Pusan National University Yangsan Hospital, Yangsan 50612, Korea; loginhhi@pnuyh.co.kr; 5 Department of Cancer Control and Population Health, National Cancer Center Graduate School of Cancer Science and Policy, National Cancer Center, Goyang 10408, Korea; 6 Rare & Pediatric Cancer Branch and Immuno-Oncology Branch, Division of Rare and Refractory Cancer, Research Institute, National Cancer Center, Goyang 10408, Korea; 7 Center for Clinical Trial, Hospital, National Cancer Center, Goyang 10408, Korea

**Keywords:** CUSUM graph, learning curve, robotic surgery, hysterectomy

## Abstract

We aim to analyze the surgical outcomes and learning curve of single-site robot-assisted hysterectomy. This was a retrospective cohort study from a single academic medical center. A total of 123 patients who underwent single-site robotic surgery for gynecologic disease were enrolled. Gynecologic surgeries were performed by a single surgeon using single-site robot-assisted hysterectomy. The median age of enrolled patients was 49 years (range: 30–74 years). The median operation time was 131 min (range: 59–502 min) and the median docking time was 3 min (range: 1–10 min). In addition, the median console time was 76 min (range: 29–465 min). The cumulative sum (CUSUM) graph for total operation time indicated an initial decrease at case 41, generating 3 distinct performance phases: learning (*n* = 41 initial cases), competence (*n* = 54 middle cases), and mastery (*n* = 28 final cases). There was one case conversion to open surgery due to the difficulty in securing the field of view because of a 16-cm bulky mass protruding from the left pelvic wall. No patients required a transfusion and two complications including vaginal cuff dehiscence were identified. The single-site robot-assisted hysterectomy is a safe and feasible procedure. The learning curve consisted of 41 cases to significantly decrease the total operation time.

## 1. Introduction

Minimally invasive surgery, including robot-assisted surgery, has been established as a new treatment option in the gynecological field over the past decade [1]. Compared to laparotomy, conventional laparoscopic surgery has become the standard surgical method due to the advantages of shorter hospital stays, reduced postoperative pain, faster recovery, lower perioperative morbidity, and improved quality of life [2]. Robotic surgery overcomes barriers of traditional laparoscopic surgery, such as the limitations of the human hand through seven degrees of movement and eliminating hand tremors. In addition, robotic arms imitate the movement of a surgeon’s hand and limit the fulcrum effect, improve visualization, and increase independence of the operating surgeon [3]. However, robotic surgery typically requires a specially trained team and may have some limitations, such as unarticulated semi-rigid instruments (i.e., harmonic scalpel device), the lack of monopolar scissors, and an unfamiliar docking process [4].

Single-site robot-assisted surgery is a relatively new concept. It is challenging because of limited movement and restricted space for multiple instruments during surgery. Literature describing a surgeon’s learning curve for robotic surgery is small in number and insufficient. Most studies included a small number of cases and multiple surgeons, and it remains unclear how many cases are required for a surgeon to reach proficiency [5,6,7]. Therefore, we implemented this study to analyze the surgical outcomes and learning curve of a single surgeon using single-site robot-assisted gynecologic surgery.

## 2. Materials and Methods

We performed a retrospective chart review of single-site robotic surgeries performed by a single surgeon (MCL) between December 2018 and October 2021 at the National Cancer Center of Korea.

All cases undergoing single-site robot-assisted gynecologic surgery were included. The surgeries included were total hysterectomy. Patient demographics, such as age, body mass index (BMI), parity, menopause, chronic illness, previous abdominal surgery, procedure types, intraoperative complication, estimated blood loss, conversion rate to open surgery, requirement of additional ports, total operation time (incision to skin closure), robotic console time, and docking time were collected. In addition, postoperative information including uterus weight, total hospital stay, readmission rate, drain insertion, and complications were recorded. The operation was performed using the da Vinci Xi Surgical System (Intuitive Surgical, Sunnyvale, CA, USA).

**Figure 1:**
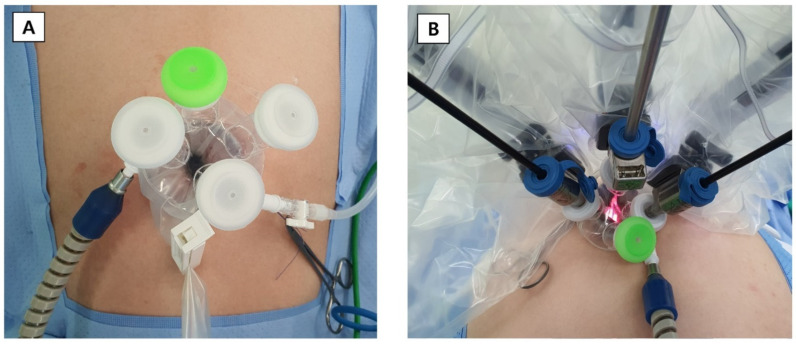
Single-site placement for robotic surgery. (**A**) Before docking. (**B**) After docking.

A 25-mm incision was made at the umbilicus. The silicon port was inserted through the transumbilical skin incision using long Kelly forceps and an Army-Navy retractor. After performing pelvic washing cytology, the uterine elevator was inserted, and the robot docking was started (Figure 1).

This study obtained the consent and approval of the Institutional Review Board (IRB) committee (IRB File No. NCC2021-0221, NCC2021-0342). The patient provided informed consent for the publication of her case including data and images.

### Statistical Analysis

The characteristics and surgical outcomes of patients who underwent singe-port robotic surgery were summarized by frequency and percent or median and range. The learning curve was analyzed by the cumulative sum (CUSUM) method using operation time. The cumulative method was used for quantitative assessment of the learning curve; it measured the running total of differences between the individual data points and mean of all data points [8]. When the operation time of each case was *X_i_* and the mean operation time was μ, CUSUM was calculated as CUSUM*_OTi_* = $\overset{n}{\underset{i=1}{\sum }}(X_{i}-\mu )$. The CUSUM*_OT1_* of the first case was the difference between the operation time for the first case and mean operation time. The CUSUM*_OT2_* of the second case was the previous case’s CUSUM*_OT1_* added to the difference between the operation time for the second case and mean operation time. Until the last case of this process, we calculated the CUSUM*_OT_* continuously.

The CUSUM curve was expressed using the CUSUM value, and the curve was divided into three phases, an increasing section, and a decreasing section using the slope. For the continuous variables, Kruskal—Wallis test was used, and the three phases were compared using Chi-squared test or Fisher’s exact test for categorical variables. The change in operation time was confirmed using the moving average method. A linear regression model was used to identify the factors affecting the operation time. Variables with *p* < 0.05 in the univariable model were included in the multivariable model, and only variables with a *p* > 0.05 remained in the model using the backward elimination method. All statistical tests were considered significant based on the significance level of *p* < 0.05 and were performed using R (version 4.0.4, The R Foundation, Vienna, Austria). 

## 3. Results

A total of 123 patients underwent single-site robot-assisted gynecologic surgery at our institution during a 35-month period by a single surgeon. The median age at the time of surgery was 49 years (range: 30–74 years) and median BMI was 23.2 kg/m^2^ (range: 18.0–34.9 kg/m^2^). Baseline characteristics of the patients and the operative characteristics are shown in Table 1.

A total of 120 (97.6%) patients underwent single-site robotic surgery for benign gynecological diseases and 3 (2.4%) patients for malignant diseases. Surgical procedures included total hysterectomy (100.0%), bilateral salpingo-oophorectomy (52.0%), ovarian cystectomy (6.5%), peritonectomy (1.6%), adhesiolysis (49.6%), pelvic washing cytology (99.2%), and pelvic lymph node dissection (15.5%) due to benign or oncologic gynecological disease in all cases. Forty-seven (38.2%) patients had undergone prior abdominopelvic surgeries and 75 (61.0%) were premenopausal patients. The most common indication for surgery was myoma (45.5%), followed by adenomyosis and myoma (22.0%).

The median operative time was 131 min (range: 59–502 min). In detail, the median docking time was 3 min (range: 1–10 min) and the median console time was 76 min (range: 29–465 min). The median estimated blood loss was 10 mL (range: 5–500 mL) and the median uterine weight was 180 g (range: 44–1230 g) (Table 2). One patient (case 118) was converted to laparotomy because of a 16-cm bulky mass protruding from the left pelvic wall and was independent of the skill level of the surgeon. No patient required an additional port to complete the procedure or received a blood transfusion. Vaginal cuff dehiscence was identified in two cases. The median total hospital stay was 4 days (range: 3–10 days).

**Figure 2:**
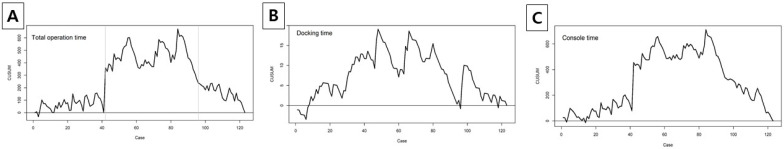
Cumulative sum (CUSUM) chart of single-site robotic surgery. (**A**) Operation time. (**B**) Docking time. (**C**) Console time.

Learning curves, regarding operation time, were obtained using CUSUM analysis. We found that the total operating time was significantly decreased after 41 cases. Three phases were observed with the learning curve of the total operation time. Based on the slope of the CUSUM chart, the analysis is divided into the learning phase 1 (*n* = 41), which shows an increasing pattern. The other two phases are the competence phase 2 (*n* = 54) and mastery phase 3 (*n* = 28), with a clear decreasing proceeded (Figure 2).

Comparing the three phases divided by operation time among patient characteristics, there were differences between the phases in chronic illness and the American Society of Anesthesiologists (ASA) classification (Table 1, *p* < 0.05). There were also statistically significant differences in incision size, estimated blood loss, drain insertion, patient-controlled analgesia use on operation day and pain killers not used in postoperative pain, and delayed postoperative complications (Table 2, *p* < 0.05). In the case of incision size, when comparing pairwise by phase, there was a significant difference between all three phases (*p* < 0.05, not attached). In the case of estimated blood loss, there were significant differences between phase 1 and the other phases (phase 1 vs. phase 2, *p* = 0.0346, phase 1 vs. phase 3, *p* = 0.0006).

Table 3 shows the linear regression results of factors that affect operation time. The univariable results revealed that operation time was significantly increased as uterine weight increased (*p* < 0.0001). In addition, operation time was significantly reduced when parity was a yes (*p* = 0.007) and when the patient had a previous abdominal surgery (*p* = 0.0385). Three factors with *p* < 0.05 in the univariable model were included in the multivariable model. Owing to backward variable selection, uterine weight and parity were statistically significant (Supplementary Figure S1).

## 4. Discussion

This study investigated the impact of a single surgeon’s robotic surgical experience on the surgical outcomes of hysterectomy utilizing a single-site robotic platform technology. The total operative time, a reflection of the learning curve with laparoscopic skills and case volume, was assessed and compared to the existing literature using the CUSUM curve.

A CUSUM curve is a graphical representation that reflects continuously performed surgical procedure trends and outcomes. Complex procedures are more likely to follow gradual learning curves, and improvement is achieved only after considerable experience. Steep learning curves imply that skills are acquired rapidly, which usually means the procedure is simple [9]. The CUSUM curve showed that operative time stabilized after gaining experience by the surgeon. It is worth emphasizing that in our study, we controlled the influence of independent variables such as BMI, concomitant procedures, previous surgeries, and group membership on the dependent variables.

In several previous studies examining hysterectomy for benign indications, greater uterine weight was associated with longer operative time [10,11,12]. Boggess et al. demonstrated this trend for robotic surgery [13]. Among all perioperative patient characteristics in their study, only uterine weight > 173.5 g was associated with increased operative time in robotic-assisted hysterectomies. In the present study, we found that 41 cases are necessary for a surgeon to become accustomed to single-site robotic surgery. In previous studies of mixed cohorts of surgeons, the learning plateau was found to be approximately 5–13 cases [14,15,16]. Peak et al. suggested that 40 cases of laparoendoscopic single-site hysterectomy were needed to achieve proficiency [17].

**Figure 3:**
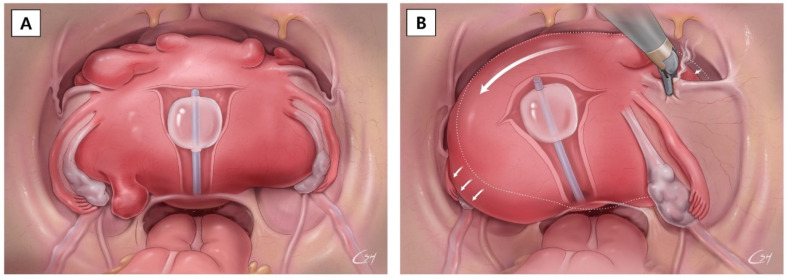
Single-site robot-assisted hysterectomy for massive mass removal. (**A**) The massive fibroid uterus fills the abdominal cavity. (**B**) Careful coagulation of round ligament using the bipolar grasper by rotation.

In our experience, robotic surgeries were feasible with a very large uterus. Regarding accessibility to the surgical target, the single-site approach is more feasible compared to a multi-port approach [18]. This is because, in recent years, the development of new technologies and instrumentation has overcome the ergonomic complexity and permits diffusion of the single-site robotic surgical approach in these cases. If the uterine mass is large, a multi-directional approach to the surgical target is difficult. It is not easy to approach the surgical target in the presence of a large tumor in the pelvis from a different direction using a multi-port robotic system. However, it is possible to perform single-port robotic surgery even if there is a large uterine tumor in the pelvis. The camera and the surgical arm can approach the surgical target from the same direction after moving the large uterine mass by pushing the uterus to the side using the RUMI^®^ II uterine elevator (CooperSurgical, Inc., Trumbull, CT, USA). Continuous cephalad pushing on the uterine elevator is protective by elevating the uterus and letting the bladder and ureters fall away from the operative field, even in the pelvic cavity with a huge uterine mass (Figure 3).

The present study has several limitations. All procedures were performed by a single robotic surgeon. Therefore, more studies including external validation is needed for the best reproducibility. The surgical learning curve was definitively related with the surgical experience of the surgeon [19]. However, we should consider a possible bias related to the different composition of assistant physicians or nursing staff. Another limitation is the retrospective study design. Lastly, additional surgical procedures with hysterectomy might be one of the confounding factors. Total operation included hysterectomy and accompanying surgical procedures. Although the additional surgical procedures including adhesiolysis or adnexal surgery do not take up a lot of proportion, this issue needs to be considered separately. Despite those limitations, our data is valuable in proving surgical feasibility and safety of single-port robotic surgery.

Our data shows that robotic single-site surgery is a feasible therapeutic option for hysterectomy. However, a large-scale and comprehensive study is required to thoroughly understand the learning curve of robotic surgery.

## 5. Conclusions

In conclusion, single-site robot-assisted hysterectomy could be an alternative treatment method to multi-port robot-assisted hysterectomy. In cases where there is a huge pelvic mass, the single-site approach can be easily accessed. From the present study, 41 cases were needed to overcome the learning curve for single-site robot-assisted hysterectomy.

## Figures and Tables

**Table 1:** Characteristics of patients who were underwent single-port robotic surgery.

	Total		Phase 1		Phase 2		Phase 3		
	(*n* = 123)		(*n* = 41)		(*n* = 54)		(*n* = 28)		
	*N*	%	*n*	%	*n*	%	*n*	%	*p*-Value
Age (yr), median (range)	49 (30–74)		50 (35–68)		48.5 (34–67)		50 (30–74)		0.8960 *
BMI (kg/m^2^), median (range)	23.2 (18.0–34.9)		22.9 (18.0–34.9)		23 (18.0–32.6)		24.4 (18.6–29.8)		0.2640 *
Parity	106	86.2	34	82.9	50	92.6	22	78.6	0.1662 ^†^
Menopause	48	39	16	39	20	37	12	42.9	0.8770 ^†^
Chronic illness	45	36.6	12	29.3	17	31.5	16	57.1	0.0359 ^†^
Vaginal delivery	73	59.4	24	58.5	32	59.3	17	60.7	0.9836 ^†^
Previous cesarean section	38	30.9	13	31.7	19	35.2	6	21.4	0.4375 ^†^
Previous abdominal surgery	47	38.2	11	26.8	19	35.2	17	60.7	0.0145 ^†^
ASA classification									<0.0001 ^‡^
I	74	60.2	33	80.5	37	68.5	4	14.3	
II	48	39	8	19.5	17	31.5	23	82.1	
III	1	0.8	0	0	0	0	1	3.6	
Indication of surgery									0.5755 ^‡^
Adenomyosis	16	13	6	14.6	8	14.8	2	7.1	
Myoma	56	45.5	20	48.8	26	48.2	10	35.7	
Adenomyosis and myoma	27	22	8	19.5	12	22.2	7	25	
Endometrial hyperplasia	1	0.8	0	0	0	0	1	3.6	
Malignancy	3	2.4	0	0	1	1.9	2	7.1	
Ovarian cyst	20	16.3	7	17.1	7	13	6	21.4	
Concomitant procedure									
Adnexectomy									0.9466 ^‡^
USO	2	1.6	1	2.4	1	1.9	0	0	
BSO	64	52	23	56.1	27	50	14	50	
Ovarian cystectomy	8	6.5	3	7.3	4	7.4	1	3.6	0.8078 ^‡^
Peritonectomy	2	1.6	1	2.4	1	1.9	0	0	1.0000 ^‡^
Adhesiolysis	61	49.6	23	56.1	28	51.9	10	35.7	0.2275 ^†^
Pelvic washing cytology	122	99.2	40	97.6	54	100	28	100	0.5610 ^‡^
Pelvic LND	19	15.5	6	14.6	9	16.7	4	14.3	0.9459 ^†^
Para-aortic LND	1	0.8	0	0	1	1.9	0	0	1.0000 ^‡^

* Kruskal—Wallis test, ^†^ Chi-squared test, ^‡^ Fisher’s exact test. Abbreviations: ASA, American Society of Anesthesiologists; BMI, body mass index; BSO, bilateral salpingo-oophorectomy; LND, lymph node dissection; USO, unilateral salpingo-oophorectomy.

**Table 2:** Surgical outcomes of single-port robotic surgery.

	Total		Phase 1		Phase 2		Phase 3		
	(*n* = 123)		(*n* = 41)		(*n* = 54)		(*n* = 28)		
	*n*	%	*n*	%	*n*	%	*n*	%	*p*-Value
Uterine mass size (cm), median (range)	6.8 (0–26)		5 (0.5–13.5)		7.5 (0–22)		9.75 (6–26)		<0.0001 *
Operation time (min), median (range)	131 (59–502)		140 (73–274)		130.5 (59–502)		129 (83–208)		0.6943 *
Docking time (min), median (range)	3 (1–10)		3 (1–6)		2 (1–10)		3 (1–10)		0.0242 *
Console time (min), median (range)	76 (29–465)		77 (41–208)		77 (29–465)		74 (40–147)		0.4023 *
Adnexal surgery	66	53.7	24	58.5	28	51.9	14	50	0.7357 ^†^
EBL (mL)	10 (5–500)		20 (5–160)		10 (5–480)		5 (5–500)		0.0007 *
Uterus weight (g)	180 (44–1230)		200.5 (50–730)		180 (44–1230)		159 (45–580)		0.2371 *
	NA = 8		NA = 5		NA = 3				
Postoperative hospital stay (days), median (range)	4 (3–10)		4 (3–6)		4 (3–10)		4 (3–7)		0.7595 *
Conversion									
Open laparotomy	1	0.8	0	0	0	0	1	3.6	0.2276 ^‡^
Drain insertion	9	7.3	7	17.1	1	1.9	1	3.6	0.0187 ^‡^
Readmission	5	4.1	1	2.4	3	5.6	1	3.6	0.8479 ^‡^
Complications									
Immediate complication									
Abdominal pain	3	2.4	0	0	3	5.6	0	0	0.3225 ^‡^
Postoperative pain (NRS)									
Use of additional pain killer: NSAIDs, Opioids	90	73.2	33	80.5	39	72.2	18	64.3	0.3216 ^†^
PCA use on operation day	11	8.9	8	19.5	3	5.6	0	0	0.0119 ^‡^
PCA use after 24 h	5	4.1	4	9.8	1	1.9	0	0	0.1188 ^‡^
Pain killer not used	28	22.8	4	9.8	14	25.9	10	35.7	0.0314 ^†^
Delayed postoperative complication									0.0296 ^‡^
Umbilical incisional hernias	3	2.4	0	0	3	5.6	0	0	
Vaginal cuff dehiscence	2	1.6	0	0	0	0	2	7.1	

* Kruskal—Wallis test, ^†^ Chi-squared test, ^‡^ Fisher’s exact test. Abbreviations: EBL, estimated blood loss; NRS, numerical rating scale; PCA, patient-controlled analgesia.

**Table 3:** Identification of factors affecting operation time using a linear regression model.

		Univariable			Multivariable (*p* < 0.05)		
		Beta	SE	*p*-Value	Beta	SE	*p*-Value
Age (yr)		−1.10	0.66	0.1012			
BMI (kg/m^2^)		2.44	1.48	0.1015			
Uterus weight (g)	Missing = 8	0.19	0.02	<0.0001	0.18	0.02	<0.0001
Parity	No	1(ref)			1(ref)		
	Yes	−51.19	14.74	0.0007	−36.49	12.42	0.0040
Menopause	No	1(ref)					
	Yes	−16.03	10.84	0.1416			
Chronic illness	No	1(ref)					
	Yes	−11.75	11.02	0.2883			
Vaginal delivery	No	1(ref)					
	Yes	−6.26	10.84	0.5646			
Previous cesarean section	No	1(ref)					
	Yes	−15.87	11.45	0.1683			
Previous abdominal surgery	No	1(ref)					
	Yes	−22.57	10.78	0.0385			
ASA classification	I	1(ref)					
	II, III	−9.95	10.86	0.3614			
Adnexectomy	None	1(ref)					
	USO, BSO	−15.47	10.60	0.1471			
Ovarian cystectomy	No	1(ref)					
	Yes	19.05	21.56	0.3788			
Other (Peritonectomy, Adhesiolysis,	No	1(ref)					
Pelvic LND, Para-aortic LND)	Yes	19.63	10.62	0.0671			

## Supplementary Materials

The following supporting information can be downloaded at: https://www.mdpi.com/article/10.3390/jcm11051378/s1, Figure S1: Linear regression graph according to parity with uterus weight (*x* axis) and operation time (*y* axis).
