# Supplementary material for: Learning Curve Analysis of Single-Site Robot-Assisted Hysterectomy
Source: J Clin Med. 2022 Mar 2;11(5):1378. doi: 10.3390/jcm11051378 (PMC8911377; doi:10.3390/jcm11051378)
Supplement: Supplementary file 1 [file jcm-11-01378-s001.zip › jcm-1568634-supplementary.pdf]

Supplementary Material

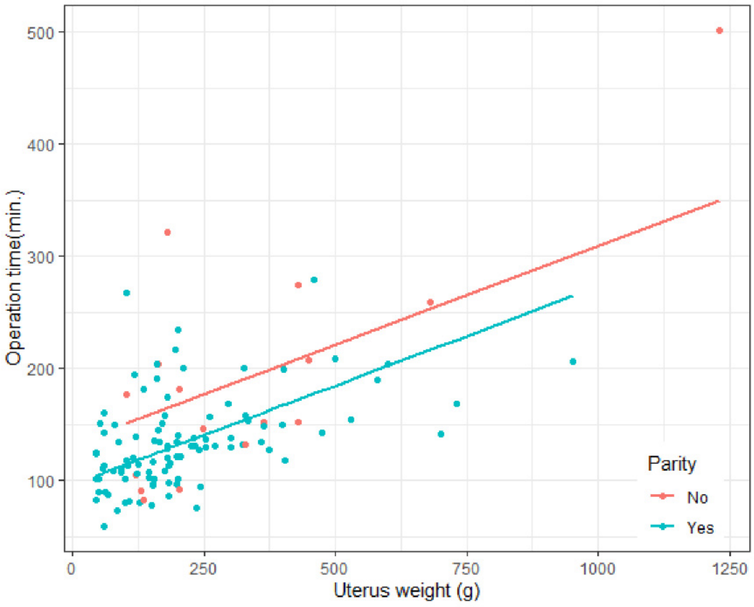

Supplementary Figure S1. Linear regression graph according to parity with uterus weight (X axis) and operation time (Y axis).
